# Improving the Accuracy of Clozapine Documentation in Primary Care Records: A Completed Audit Cycle Within a Community Mental Health Team

**DOI:** 10.1192/bjo.2026.11554

**Published:** 2026-06-30

**Authors:** Rizwan Anwer, George Mosa

**Affiliations:** Cornwall Foundation and Partnership Trust, Cornwall, United Kingdom

## Abstract

**Aims:**

Clozapine is a high-risk antipsychotic prescribed in secondary care, requiring accurate documentation in primary care records to ensure patient safety and continuity of treatment. Inaccurate recording of Clozapine dose and frequency may result in prescribing errors, treatment interruption, or adverse outcomes, particularly in emergency or out-of-area settings. An initial audit identified significant deficiencies in the accuracy of Clozapine dose and frequency documentation within primary care records for patients managed by a community mental health team.

**Methods:**

An audit was conducted within the North Cornwall Community Mental Health Team assessing the accuracy of Clozapine documentation in primary care records. Standards were derived from the Cornwall Partnership NHS Foundation Trust “Procedure and Guidance for the Use of Clozapine” (MM/032/22). Following dissemination of findings and targeted liaison with GP practices, a re-audit was undertaken using the same patient cohort (n=15) and methodology. Summary Care Records were reviewed and compared against the most recent secondary care clinic correspondence to assess inclusion of Clozapine on GP repeat prescriptions and accuracy of documented dose and frequency.

**Results:**

In the initial audit, Clozapine was included on GP repeat prescriptions in 93% of cases; however, accurate documentation of dose and frequency was present in only 33%. The re-audit demonstrated sustained inclusion of Clozapine on repeat prescriptions (93%) alongside a substantial improvement in accurate dose and frequency documentation, rising to 80%. These findings indicate a marked improvement following intervention, representing a meaningful enhancement in patient safety. Residual inaccuracies persisted in a minority of cases, highlighting the ongoing importance of clear communication between secondary and primary care.

**Conclusion:**

Completion of the audit cycle demonstrated that targeted intervention and improved liaison with GP practices significantly enhanced the accuracy of Clozapine dose documentation in primary care records. While overall documentation improved substantially, continued vigilance and clear communication following medication changes remain essential to minimise prescribing risk. Ongoing monitoring is recommended to sustain these improvements and ensure patient safety.

